# Decomposition and decoupling analysis between economic growth and carbon emissions at the regional level: Evidence from six central provinces, China

**DOI:** 10.1371/journal.pone.0305769

**Published:** 2024-09-06

**Authors:** Wensheng Wang, Xuanyi Zhu, Xiaoxuan Kao, Hui Wei

**Affiliations:** 1 School of Management, China University of Mining and Technology-Beijing, Beijing, China; 2 Research Institute of Decision-making Science and Big Data, China University of Mining and Technology-Beijing, Beijing, China; National Center for Chronic and Non-Communicable Disease Prevention and Control, CHINA

## Abstract

As the six central provinces account for 23% of total national carbon emissions (CE), research into the decoupling status of their economic growth (EG) and carbon emissions is critical to achieving the Dual Carbon Goals and the Rise of Central China Plan. This research initially examines the decoupling status between CE and EG using the Tapio decoupling model, based on energy consumption (EC) dataset from six central provinces in China between 2000 and 2019. The decoupling index (DI) is then divided into five decoupling drivers using the LMDI method. Finally, an enhanced STIRPAT model is used to examine the decoupling status of CE and EG in the six central provinces from 2020 to 2040. The research findings are: (1) The six central provinces exhibited a stable decoupling status between 2000 and 2019. The DI of the six central provinces ranged from -1.2 to 3.4. (2) The decoupling performance is influenced mainly by the inhibitory effect of economic development (GI) and the promoting effect of energy intensity (EI). The GI consistently maintains an impact value of around 0.9. EI performance varies widely across provinces. (3) From 2020 to 2040, Anhui, Hubei, Henan, and Hunan show significantly strong decoupling indices distributed between -2.21 and -0.07 in all three scenarios. It is important to note that Shanxi and Jiangxi provinces will experience a Reverse Decoupling phenomenon. These findings are helpful in developing regionally coordinated development policies and strategies for reducing CE.

## Introduction

China has witnessed tremendous economic expansion since the reform and opening up, which has resulted in a constant increase in EC. China’s EC and CE have surpassed the United States [1]. China made a significant commitment to reduce its carbon intensity (CI) by more than 65% by 2030 at the 75th United Nations General Assembly. Furthermore, China aims to increase the share of non-fossil energy to approximately 25% [2]. Overall, it is inevitable that the growth of the Chinese economy will no longer be dependent on the consumption of fossil fuels [3]. Therefore, exploring the relationship between EG and CE is significant in facing the pressures of the Dual Carbon Goals and economic development. Decoupling analysis is one of the most effective methods for measuring the relationship between them. However, proper analysis of the EG and EC’s decoupling status and influencing variables is the foundation for developing emission reduction strategies. Accelerating the decoupling process between EG and CE has also been an essential priority in recent years [4].

Numerous scholars have undertaken studies based on the preceding background. The scholars’ research has centered on three major areas. The first field is to investigate the elements that influence decoupling. The influencing factors are classified into several categories determined by the research subject. The main factors under consideration are energy structure (EG), energy efficiency (EI), economic development level (GI), population (P), etc. EG is the main factor affecting CE [5,6]. The second area is investigated from sectoral perspectives, primarily in the industrial, agricultural, and transportation sectors. Some findings suggest that China’s power sector has decoupled, but it still shows weak decoupling, and the industry still has significant potential for future emission reductions [7,8]. Lastly, research that examines issues from a national or local perspective also exists [9]. For example, Fang et al. analyzed the decoupling status of 30 provinces in China and projected the peak time of CE. The results show that most provinces will achieve peak CE before 2030 [10]. Jia et al. analyzed the factors impacting household CE in six central provinces, and the findings revealed a significant disparity across the six provinces, which is gradually diminishing. Optimizing the ES and improving people’s consumption patterns will be critical in the future [11].

In conclusion, past research has laid some groundwork, but significant gaps remain. For starters, fewer studies focus on CE from the consumption of energy. However, CO_2_ from EC makes up a significant share of total CE. To accurately assess the process of meeting the Dual Carbon Goals, it is critical to examine the decoupling status of CE from EC and EG. Second, there needs to be more analysis from a regional perspective, especially in the six central provinces. This paper believes that regional studies are critical for achieving national decoupling. The six central provinces, namely Anhui, Shanxi, Hubei, Henan, Hunan, and Jiangxi, possess strategic geographical locations, connecting vital regions such as the “Bohai Rim” in the north, the “Pearl River Delta” in the south, and the “Yangtze River Delta” in the east. The EC of the six central provinces represents almost 23% of the total. They are essential energy consumers. Besides, the six central provinces are facing a rapid rise in CE [12]. If the six core provinces’ decoupling status is not precisely examined, the gap between them and the other provinces will expand, making it challenging to meet the Dual Carbon Goals. Finally, no literature has been published on predicting the decoupling status. The majority of research estimates the peak time of CE. However, peaking CE does not guarantee decoupling, so it is required to estimate the decoupling status.

Therefore, we intend to investigate the decoupling status of CE and EG in the six central provinces from various perspectives, as well as forecast the decoupling status for the period 2020–2040, which will ultimately create an overall analytical framework for the decoupling process in the six central provinces. The main question can be broken down into three smaller questions. 1. What is the status of decoupling between CE and EG in the six central provinces during 2000–2019? 2. What factors influence the decoupling status? 3. Will all six provinces be able to achieve a strong decoupling of CE and EG in the next two decades? To answer these questions, the Tapio model is used to assess the decoupling status of the six central provinces between 2000 and 2019, laying the groundwork for future investigations of affecting factors. Introducing the LMDI model in combination with the Tapio model to identify the contributing elements that generate these decoupling statuses. The STIRPAT model is used to estimate the decoupling status of the six central provinces from 2020–2040 under three different scenarios, which will provide an important reference for the provinces to formulate strategies.

**Fig 1:**
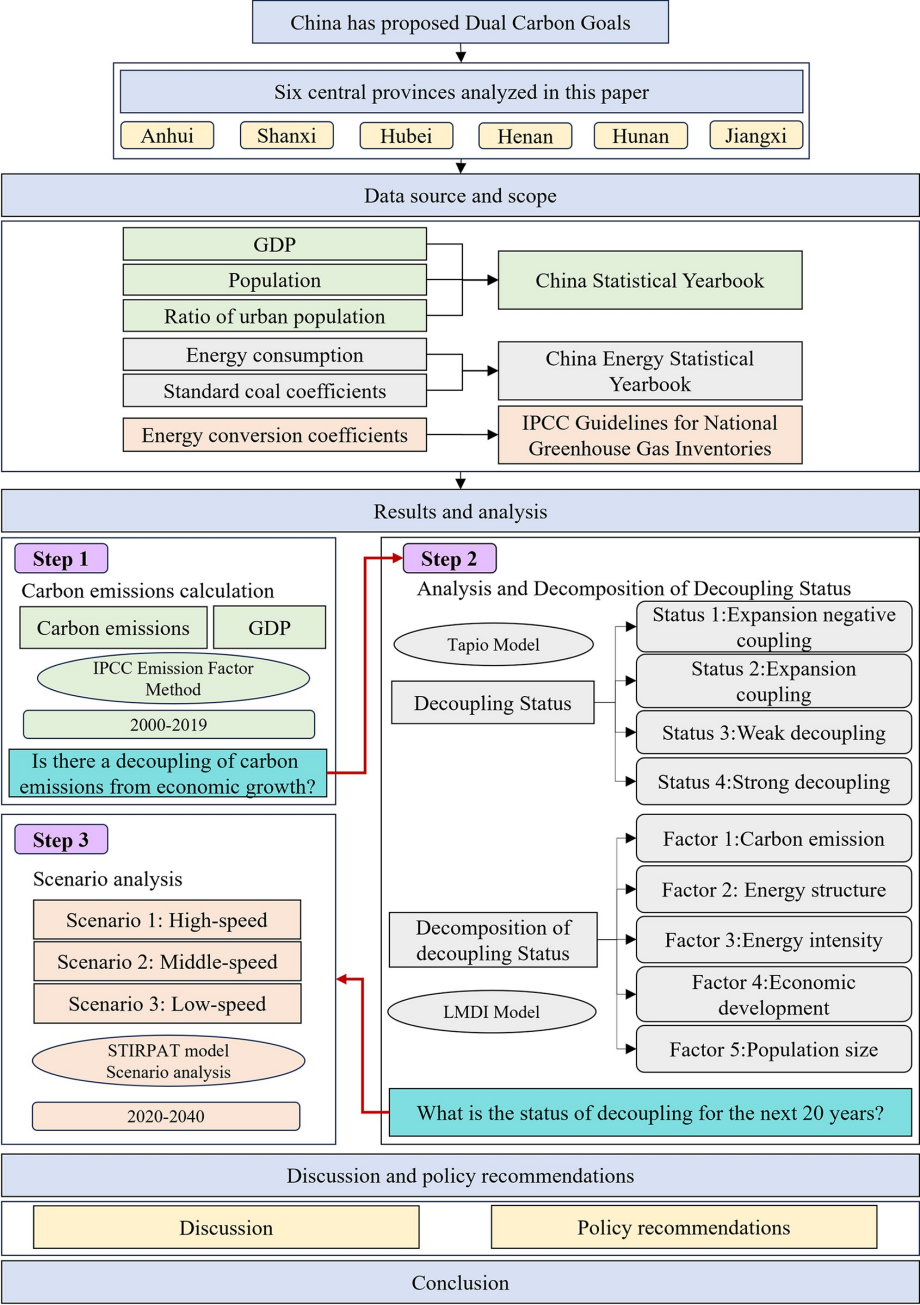
Framework diagram for this paper.

The remainder of this paper consists of six sections that are organized as follows: Section 2 reviews the relevant literature; Section 3 introduces the data sources and research methods; Section 4 displays the experimental results and provides in-depth analyses of the results; Section 5 makes some informative recommendations for provinces’ future development; and Section 6 summarizes the entire text. The framework diagram for this paper is shown in Fig 1.

## Literature review

There are three main categories related to CE among scholars. The first category mainly studies the relationship between CE and EG. The second category mainly studies the driving factors that affect CE. The third category focuses on predicting the peak time of CE.

**Fig 2:**
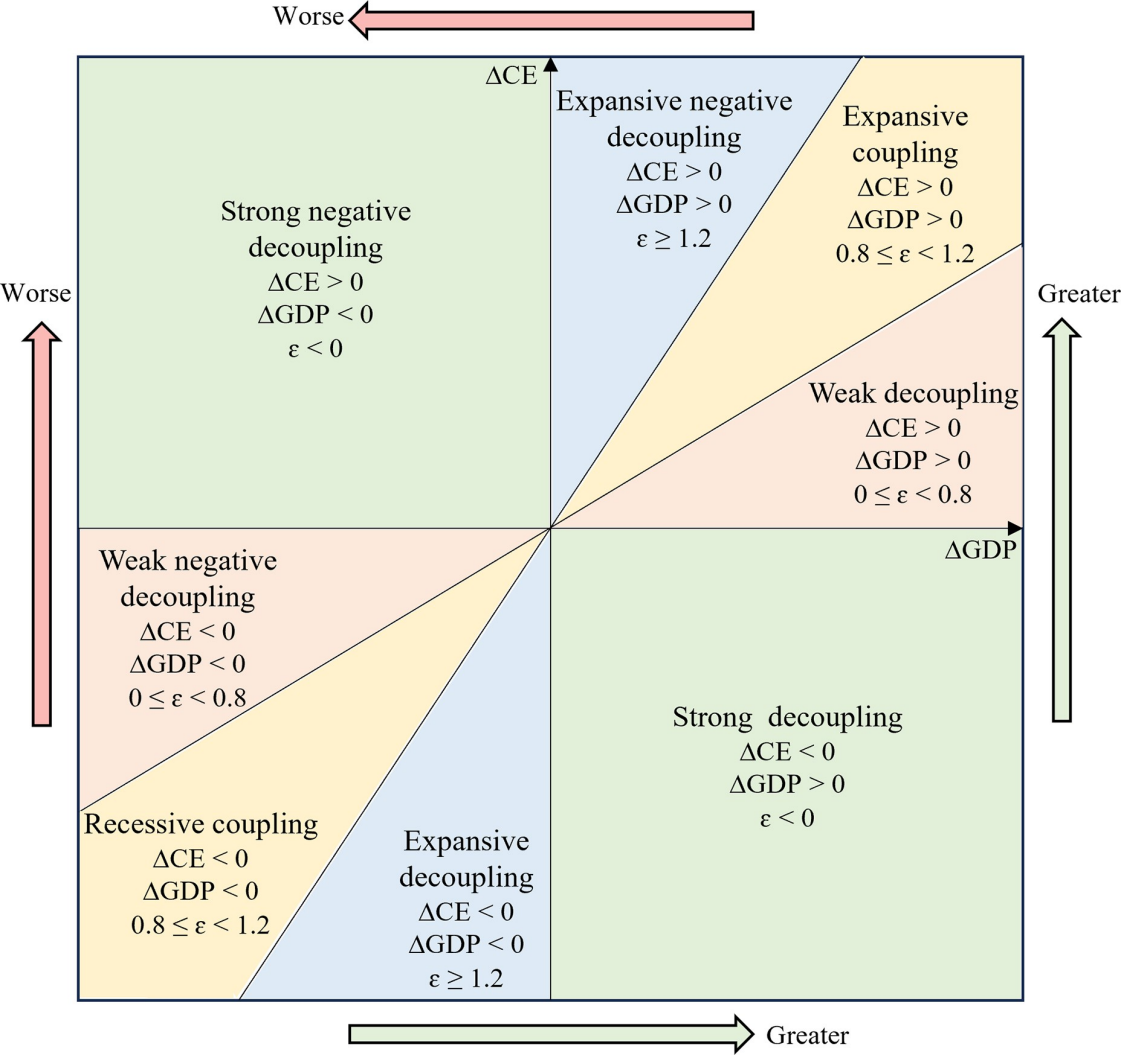
Classification criteria for decoupling status.

EG has a significant influence on CE, and the relationship between them is of important scholarly interest. Overall, the relationship between CE and EG can be divided into two types, which include correlation and decoupling relationships. Decoupling measures the coupling rupture relationship between human activities and resource environmental pressures. The decoupling of EG and CE is necessary to achieve the Dual Carbon Goals. According to the OECD and Tapio’s taxonomy, there are eight major types of decoupling status, as indicated in Fig 2 [13]. In recent years, much literature has applied the Tapio model to study decoupling in different regions, industries, or countries [14]. The Chinese provinces are in a stable state of decoupling, but the pace is gradual and the outcomes are unsatisfactory [15]. Some researchers have used the Tapio model to investigate the decoupling status of EG and CE in the industry. For example, Jia et al. discovered that China’s agricultural land use has been effectively managed, with a transition from a weak decoupling status before 2015 to a strong decoupling status after that [16]. Jiang et al. concluded that per capita arable land area and rural population are the main factors influencing CE in the Chinese agricultural sector, and there are six decoupling statuses between CE and agricultural output [17]. In addition, a number of researchers have used the approach to study the decoupling status of other countries. Hu et al. analyzed the temporal and spatial evolution of CE decoupling in countries along “The Belt and Road” from 1991 to 2016 [18]. They discovered that high-income countries decoupled faster than low-income countries. These successful experiments demonstrate that the Tapio model is a useful instrument for assessing the status of decoupling economic growth from carbon emissions.

In addition to EG, other factors have an impact on CE. Scholars have commonly employed the factor decomposition analysis method to investigate the factors that influence CE comprehensively. Structural decomposition analysis (SDA) and exponential decomposition analysis (IDA) are the two main decomposition methods [19,20]. SDA is a direct or indirect study of CE based on input-output tables. Because of slow data updates, this method is less commonly used [21]. Compared with SDA, IDA is widely used [22,23]. IDA consists of Laspeyres’ exponential decomposition and Divisia’s exponential decomposition [24]. The first decomposition method has yet to be widely used due to its inability to decompose all factors [25]. The second method is categorized into the Arithmetic Mean Divisia Index (AMDI) and Logarithmic Mean Divisia Index (LMDI) methods [26]. Since AMDI cannot solve the zero-value problem, many scholars have used the LMDI [27]. There are many successful examples of the applicability of the LMDI model to study different industries or countries [28]. The LMDI model theoretically demonstrates that EG is the primary factor determining CE. He et al. used the LMDI method to decompose the CE factors in China’s power industry. Their results showed that EG is the main driving factor for CE [29]. The impact of other elements is not overlooked. Some scholars have also applied the combination of LMDI and Tapio models to analyze the decoupling status and decomposition of factors in different industries in some provinces [30–35]. The factors are heterogeneous across industries and regions. Jia et al. investigated the current state of CE and influencing factors in China’s tourism industry and discovered that tourist scale, tourism consumption, and sectoral structure are the primary contributors to CE. In contrast, EI and spatial distribution structure are the primary inhibitors of CE [36]. Jin et al. studied the decoupling status of China’s manufacturing industry and found that the main factors affecting CE are fixed asset investment and investment in CI [37]. Pan et al. studied 29 provinces in China and found that technological progress is the key factor [38]. Liu et al. argued that CI and innovation inputs are critical determinants of the decoupling status in manufacturing [39]. Wang et al. studied the decoupling status of the transportation industry in Zhejiang province and its influencing factors [40]. Wen et al. studied the decoupling status and influencing factors of the energy industry in Liaoning Province [41]. They found that EG and ES are factors that promote industrial CE, while EI and technology are factors that suppress industrial CE. Fatima et al. decomposed the drivers of industrial CE in China from 1991–2016 using the LMDI methodology, which showed that income and labor were the most critical factors affecting CE. Meanwhile, many scholars have utilized the LMDI to study different countries [42]. For example, Simbi et al. studied the factors affecting CE in African countries and found that population and EG are the main drivers of CE [43]. In summary, most scholars decompose the factors affecting CE into five factors: carbon intensity (CI), energy structure (ES), energy intensity (EI), economic development (GI), and population size (P), which are used in this paper.

As the largest developing country, China’s EC and CE are still on an upward trend as urbanization continues. Therefore, predicting the peak time of CE is particularly important. Because of the disparities in development between provinces and industries, a number of scholars have investigated when and how much peak carbon emissions occur in specific provinces or industries. In the early years, STIRPAT was used for the decomposition simulation of influencing factors, which was employed by Jia et al. to research the influencing factors of the ecological footprint of Henan Province from 1983 to 2006, and discovered that population, GDP per capita, and so on, were the significant contributor [44]. The model has been improved in recent years, and scholars are beginning to use it for prediction. Zhao et al. used the STIRPAT model to analyze household CE footprints and CE peak times in 30 provinces of China. The results indicate that 25 provinces can peak household carbon emissions [45]. Wei et al. used the STIRPAT model to predict the CE of Henan Province from 2020 to 2040 [46]. Li et al. projected CE in the construction sector from 2018 to 2045 using scenario analysis, which showed that China’s construction sector would peak in 2045 at the latest and in 2020 at the earliest [47]. Gu et al. projected CE in Shanghai from 2016–2030 and projected that Shanghai’s CE would peak in 2025 [48]. In addition to the STIRPAT model and scenario analysis methodologies, scholars have employed a variety of other models to forecast. Wang et al. used the Monte Carlo model to simulate the peak time of CE and predicted that CE would reach its peak between 2021 and 2025 [49]. Huang et al. used the grey correlation to establish the Long-short-term memory (LSTM) method to predict CE [50]. In general, these literatures have designed different scenarios under various economic and technological conditions and projected CE at various levels and in different industries. However, there needs to be more literature on projections of the decoupling status of CE. The peak time of CE does not imply that decoupling status has been achieved. If simply the peak time is used as a criterion for judgment, provincial governments may overestimate the process of achieving the Dual Carbon Goals, which is detrimental to their smooth implementation.

Previous investigations have built a solid foundation. While the existing research mainly focuses on a national perspective or specific provinces, it lacks a regional perspective [51]. China has a vast landmass with considerable regional variances in development levels. Significant variances exist across provinces or regions regarding resource endowment, economic organization, and technological advancement [52,53]. Achieving the national Dual Carbon Goals requires joint efforts from all regions, so studying their decoupling status from a regional perspective is essential. Furthermore, previous studies have primarily focused on the peak time of CE. However, there is no substantial positive relationship between peak time and decoupling status. If policy design is based purely on the peak time of CE, it is bound to be skewed. Therefore, the prediction of the decoupled status is essential. According to earlier literature, the Tapio model, LMDI model, and STIRPAT model have been successfully used in relevant investigations. Thus, this paper will combine the LMDI and Tapio methods to analyze the decoupling status of CE in the six central provinces of China and use the scenario analysis method to predict their decoupling status in 2020–2040.

The main contributions of this paper are as follows: 1. In contrast to earlier research, this paper focuses on the decoupling analysis of energy-based CE, which is more important to regions with high EC, like the six central provinces. This can address a gap in the decoupling analysis of six central provinces from the standpoint of the region and serve as a theoretical foundation for the formulation of CE reduction policies in these provinces. 2. Unlike prior studies, this paper enriches the factors affecting CE, emphasizes the importance of population and urbanization, and improves policy formulation accuracy. 3. In contrast to earlier studies that projected the peak time of CE, this paper provides a new perspective to predict the decoupling status of the six central provinces over the next 20 years, which was not available in previous studies. The research findings in this paper provide unique insights and crucial references for decoupling policies in six central provinces and will give valuable opinions and recommendations to help the country meet the Dual Carbon Goals.

## Data sources and methodology

### Data sources

This paper takes 2000–2019 as its interval and uses coal, oil, and natural gas as objects to study the decoupling status of CE and EG in six central provinces. Gross Domestic Product (GDP) data is utilized to measure EG. In order to enhance data comparability, the GDP figures are adjusted by using the year 2000 as the benchmark year. Data on EC and their conversion coefficients to standard coal are gathered from the China Energy Statistical Yearbook. In terms of CE, this paper relies on the CE coefficient sourced from the IPCC report. The specific data is shown in Table 1.

**Table 1:** Coefficients of conversion to standard coal and CE.

Energy Type		Coefficient of conversion to standard coal (kgce/kg)	Coefficient of CE (CO_2_/ kg)
Coal	Crude Coal	0.714	2.690
	Coke	0.971	2.690
Oil	Crude Oil	1.429	2.760
	Gasoline	1.471	2.925
	Kerosene	1.471	3.018
	Diesel	1.457	3.096
	Fuel Oil	1.429	3.171
Gas	Natural Gas	1.200 kgce/m^3^	2.162 CO_2_/ m^3^

### Methodology

#### Carbon emission calculations

According to the IPCC guidelines, a method based on the total amount of carbon in the fuel is used to estimate CE. The calculation method for CE during the period t is shown in Formula (1).

$$CE_{i,t}=\frac{44}{12}\sum_{j=1}^{3}E_{j,t}\times a_{j}\times \beta_{j}$$ (1)

where *E*_*j*,*t*_ is the physical consumption of energy source *j* in period *t*, *a*_*j*_ is the discounted standard coal factor of energy source *j*, and *β*_*j*_ represents the CE factor of energy source *j*. The CE factor is 44/12. Various energy types, such as crude coal, coke, crude oil, gasoline, kerosene, diesel, fuel oil, and natural gas, are considered.

#### Tapio and LMDI model

The model in this paper is based on Tapio (2005), which calculates the decoupling index (DI) between CE and EG, as shown in Eq (2).

$$\varepsilon {\left(CE,GDP\right)}_{t}=\frac{\%\Delta CE_{i,t}}{\%\Delta GDP_{i,t}}=\frac{\Delta CE_{i,t}/CE_{i,0}}{\Delta GDP_{i,t}/GDP_{i,0}}$$ (2)

*ε(CE*,*GDP)*_t_ indicates the DI between CE and EG from year *(t-1)* to year *t*. %*ΔCE*_*i*,*t*_ indicates the rate of change of CE from a particular base period to year *t*. *CE*_*i*,*0*_ is the CE in the base year. *ΔCE*_*i*,*t*_ indicates the change in CE from the base period to year *t*. %*ΔGDP*_*i*,*t*_ is the rate of change in GDP. *GDP*_*i*,*0*_ indicates the GDP value for the base year and *ΔGDP*_*i*,*t*_ denotes the change in GDP from the base year to year *t*.

According to Kaya’s equation, the CE drivers are decomposed into carbon intensity (CI), energy structure (ES), energy intensity (EI), economic development (GI), and population size(P), as shown in Eq (3).


$$CE_{t}=\sum_{j=1}^{3}\frac{CE_{j,t}}{E_{j,t}}\times \frac{E_{j,t}}{E_{t}}\times \frac{E_{t}}{GDP_{t}}\times \frac{GDP_{t}}{P_{t}}\times P_{t}=CI_{j,t}\times ES_{j,t}\times EI_{t}\times GI_{t}\times P_{t}$$ (3)

*CE*_*t*_ is the total CE at time *t*. *E*_*j*,*t*_ is the EC of energy source *j* at time *t*. *E*_*t*_ denotes the total emissions from all energy at time *t*. *GDP*_*t*_ denotes the value of GDP in a province at time *t*. *P*_*t*_ indicates the population of a province at time t. *CI*_*j*,*t*_ denotes the CI of energy *j* at time *t*. *ES*_*j*,*t*_ denotes the ES of energy source *j* at time *t*. *EI*_*t*_ denotes the EI at time *t*. *GI*_*t*_ indicates the level of economic development at time *t*. *P*_*t*_ denotes the level of population size at time *t*.

From the additive form of LMDI, the amount of CE change can be decomposed into the sum of five major factors: *ΔCI*, *ΔES*, *ΔEI*, *ΔGI*, and *ΔP*, as shown in Eq (4).


$$\Delta CE_{i,t}=CE_{i,t}‐CE_{i,0}=\Delta CI+\Delta ES+\Delta EI+\Delta GI+\Delta P$$ (4)

The factors are calculated from Eq (5).


$$\begin{array}{l} \Delta CE_{ci}=\left\{ \begin{array}{c} 0,if\;CE_{j,t}\times CE_{j,0}=0\\ \underset{j}{\Sigma }\frac{CE_{j,t}‐CE_{j,0}}{In(CE_{j,t}/CE_{j,0})}\times In(CI_{j,t}/CI_{j,0}),if\;CE_{j,t}\times CE_{j,0}\neq 0 \end{array}\right.\\ \Delta CE_{es}=\left\{ \begin{array}{c} 0,if\;CE_{j,t}\times CE_{j,0}=0\\ \underset{j}{\Sigma }\frac{CE_{j,t}‐CE_{j,0}}{In(CE_{j,t}/CE_{j,0})}\times In(ES_{j,t}/ES_{j,0}),if\;CE_{j,t}\times CE_{j,0}\neq 0 \end{array}\right.\\ \Delta CE_{gi}=\left\{ \begin{array}{c} 0,if\;CE_{j,t}\times CE_{j,0}=0\\ \underset{j}{\Sigma }\frac{CE_{j,t}‐CE_{j,0}}{In(CE_{j,t}/CE_{j,0})}\times In(GI_{t}/GI_{0}),if\;CE_{j,t}\times CE_{j,0}\neq 0 \end{array}\right.\\ \Delta CE_{p}=\left\{ \begin{array}{c} 0,if\;CE_{j,t}\times CE_{j,0}=0\\ \underset{j}{\Sigma }\frac{CE_{j,t}‐CE_{j,0}}{In(CE_{j,t}/CE_{j,0})}\times In(P_{t}/P_{0}),if\;CE_{j,t}\times CE_{j,0}\neq 0 \end{array}\right.\\ \Delta CE_{ei}=\left\{ \begin{array}{c} 0,if\;CE_{j,t}\times CE_{j,0}=0\\ \underset{j}{\Sigma }\frac{CE_{j,t}‐CE_{j,0}}{In(CE_{j,t}/CE_{j,0})}\times In(EI_{t}/EI_{0}),if\;CE_{j,t}\times CE_{j,0}\neq 0 \end{array}\right. \end{array}$$ (5)

The Tapio decoupling model and the LMDI model are combined to decompose the decoupled status as shown in Eq (6).


$$\begin{array}{l} \varepsilon {\left(CE,GDP\right)}_{t}\\ =\frac{\Delta CE_{i,t}/CE_{i,0}}{\Delta GDP_{i,t}/GDP_{i,0}}\\ =\frac{(\Delta CI+\Delta ES+\Delta EI+\Delta GI+\Delta P)/CE_{i,0}}{\Delta GDP_{i,t}/GDP_{i,0}}\\ =\frac{\Delta CI\times GDP_{i,0}}{CE_{i,0}\times \Delta GDP_{i,t}}+\frac{\Delta ES\times GDP_{i,0}}{CE_{i,0}\times \Delta GDP_{i,t}}+\frac{\Delta EI\times GDP_{i,0}}{CE_{i,0}\times \Delta GDP_{i,t}}+\frac{\Delta GI\times GDP_{i,0}}{CE_{i,0}\times \Delta GDP_{i,t}}+\frac{\Delta P\times GDP_{i,0}}{CE_{i,0}\times \Delta GDP_{i,t}}\\ =\varepsilon_{ci}+\varepsilon_{es}+\varepsilon_{ei}+\varepsilon_{gi}+\varepsilon_{p} \end{array}$$ (6)

*ε*_*ci*,_ *ε*_*es*,_ *ε*_*ei*,_ *ε*_*gi*,_ and *ε*_*p*_ are the elasticities of the corresponding drivers, and the decoupling elasticity of CE from EC can be decomposed into the sum of the decoupling elasticities of the five drivers.

#### STIRPAT model

Ehrlich et al. presented the IPAT model, on which York based the STIRPAT model [54,55]. The model expression is shown in Eq (7).

$$I=\alpha P^{\beta }A^{\theta }T^{\gamma }e$$ (7)

where *I*, *P*, *A*, and *T* are environmental pressures, population size, affluence, and technology level. *α* is the model coefficient, *β*, *θ*, and *γ* are the indexes of each variable, and *e* is the model error. STIRPAT is a nonlinear model with multiple independent variables. The natural logarithm is taken from the above equation, and the STIRPAT model is expanded in this paper. The equation is shown in Formula (8).


InI=Inα+βInP+θInA+γInT+ωInU+Ine (8)

*InI* is the CE generated by EC. *Ina* is a constant term. *Ine* is the error term. *βInP* indicates the extent to which changes in population size affect CE. *θInA* indicates the extent to which changes in GDP per capita affect CE. *InT* indicates EI, characterized by the ratio of EC to GDP. *InU* indicates the level of urbanization, characterized by the ratio of urban population to resident population. Where *β*, *θ*, *γ*, and *ω* denote the elasticity coefficients of each variable.

## Results and analysis

### Analysis of CE and economic aggregate

**Fig 3:**
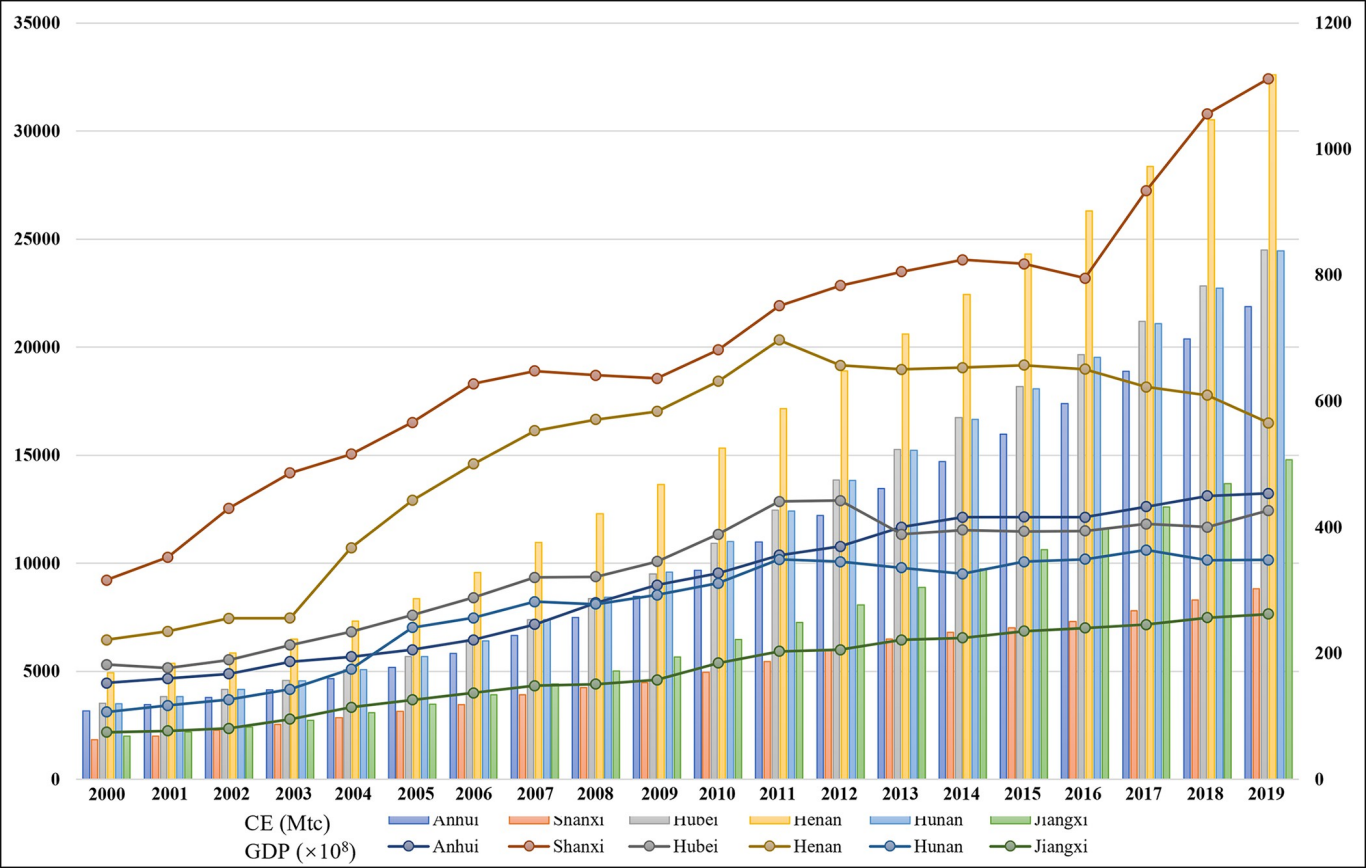
CE and EG of the six central provinces.

As shown in Fig 3, CE in the six central provinces has witnessed a substantial surge from 1054 Mtc in 2000 to 3168 Mtc in 2019. Specifically, the average annual growth rate of CE between 2000 and 2005 stood at 12%, which decreased to 7% from 2006 to 2010. Notably, a significant decline was observed from 2011 to 2019, with a rate of merely 3%. The average annual growth rate of CE in the six central provinces has tended to decrease continuously over the years. This tendency helps to achieve decoupling status. Considering individual provinces, the total CE of the six central provinces is ranked as follows: Shanxi > Henan > Anhui > Hubei > Hunan > Jiangxi. There is a significant difference in CE across provinces with the highest and lowest levels, and the reasons for this disparity must be researched further. Fig 3 also provides insights into the GDP growth of each province over the past two decades. The average EG rate amounted to 12% between 2000 and 2005, 13% between 2006 and 2010, and 10% from 2011 to 2019. Notably, implementing of the Rise of Central China Strategy in 2006 has fueled the EG’s rapid growth, contributing to the aforementioned trends. In terms of provinces, the total EG is ranked as follows: Henan > Hubei > Hunan > Anhui > Jiangxi > Shanxi. Overall, the total EG and CE of the six central provinces showed an increasing trend. However, the optimum situation for decoupling is when EG is no longer dependent on CE. It is uncertain whether the six central provinces have achieved this status, further investigation is warranted.

### Analysis and decomposition of decoupling status

#### The decoupling results

**Fig 4:**
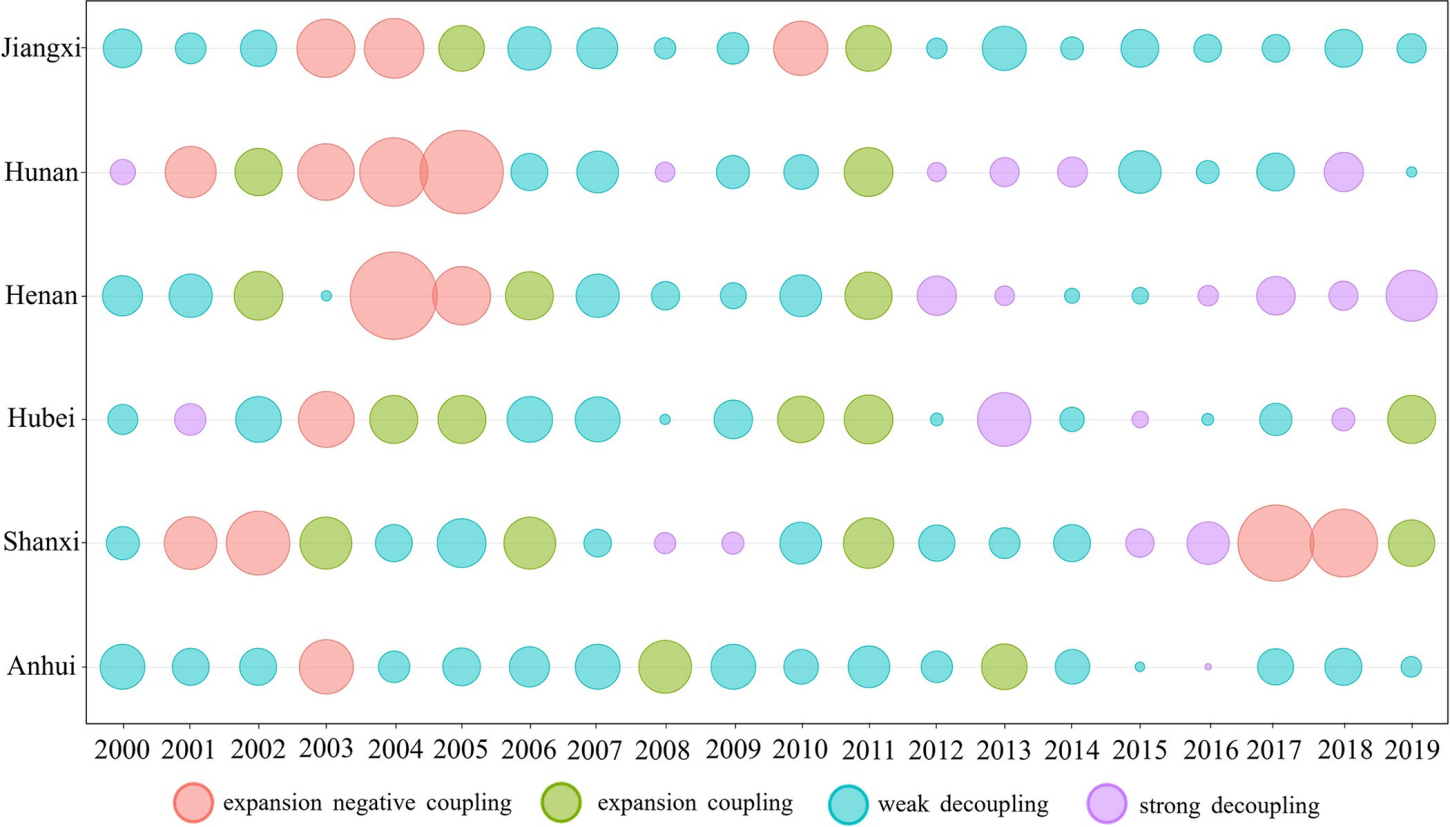
Decoupling status of six central provinces.

Fig 4 depicts the decoupling status between CE and EG in the six central provinces from 2000 to 2019. Considering that all the EG rates recorded positive values, four distinct decoupling statuses can be observed, namely: expansion negative coupling (I), expansion coupling (II), weak decoupling (III), and strong decoupling (IV). In order to analyze the decoupling relationship between CE and EG in the six central provinces, this paper is separated into four subperiods based on the division of the five-year plan: 2000–2005, 2006–2010, 2011–2015, and 2016–2019.

According to Fig 4, the DI of the six central provinces ranged from -0.3 to 3.1 between 2000 and 2005. During this period, the decoupling performance of the six central provinces was insignificant, with the primary decoupling status being weak decoupling. Anhui Province briefly experienced an expansion of the negative coupling status in 2003, while the remaining years demonstrated a weak decoupling status, which is in keeping with the findings of Chen et al. [56]. Shanxi Province exhibited weak decoupling for three years. Hubei, Henan, Hunan, and Jiangxi provinces showcased relatively weak decoupling performance, which is similar to the findings of Chun et al. [57]. In summary, Anhui province displayed the most favorable decoupling performance during the 10th five-year period. During the 11th five-year period, the decoupling performance of the six central provinces improved significantly. Anhui Province maintained its decoupling status during the 11th five-year plan period. Shanxi Province showed strong decoupling for two consecutive years. Hubei Province and Hunan Province showed stable weak decoupling, and Jiangxi Province experienced an expansion of negative coupling status in only one year, while the remaining years showcased weak decoupling. Overall, during the 11th five-year plan period, the decoupling status of the six central provinces notably improved. During the 12th and 13th five-year, the EG rate in the six central provinces exhibited a slowdown. However, despite this slowdown, the decoupling status of most provinces still showcased a favorable performance. Hunan Province has demonstrated the best performance, consistently maintaining a strong decoupling status since 2016. Overall, the decoupling status of the six central provinces has shown significant improvement since 2006, with most provinces exhibiting a stable decoupling status. However, Shanxi Province’s decoupling performance stands out, demonstrating both expansion negative coupling and expansion coupling between 2017 and 2019, leading to a unique Reverse Decoupling phenomenon that deserves further research.

#### The decomposition results

**Fig 5:**
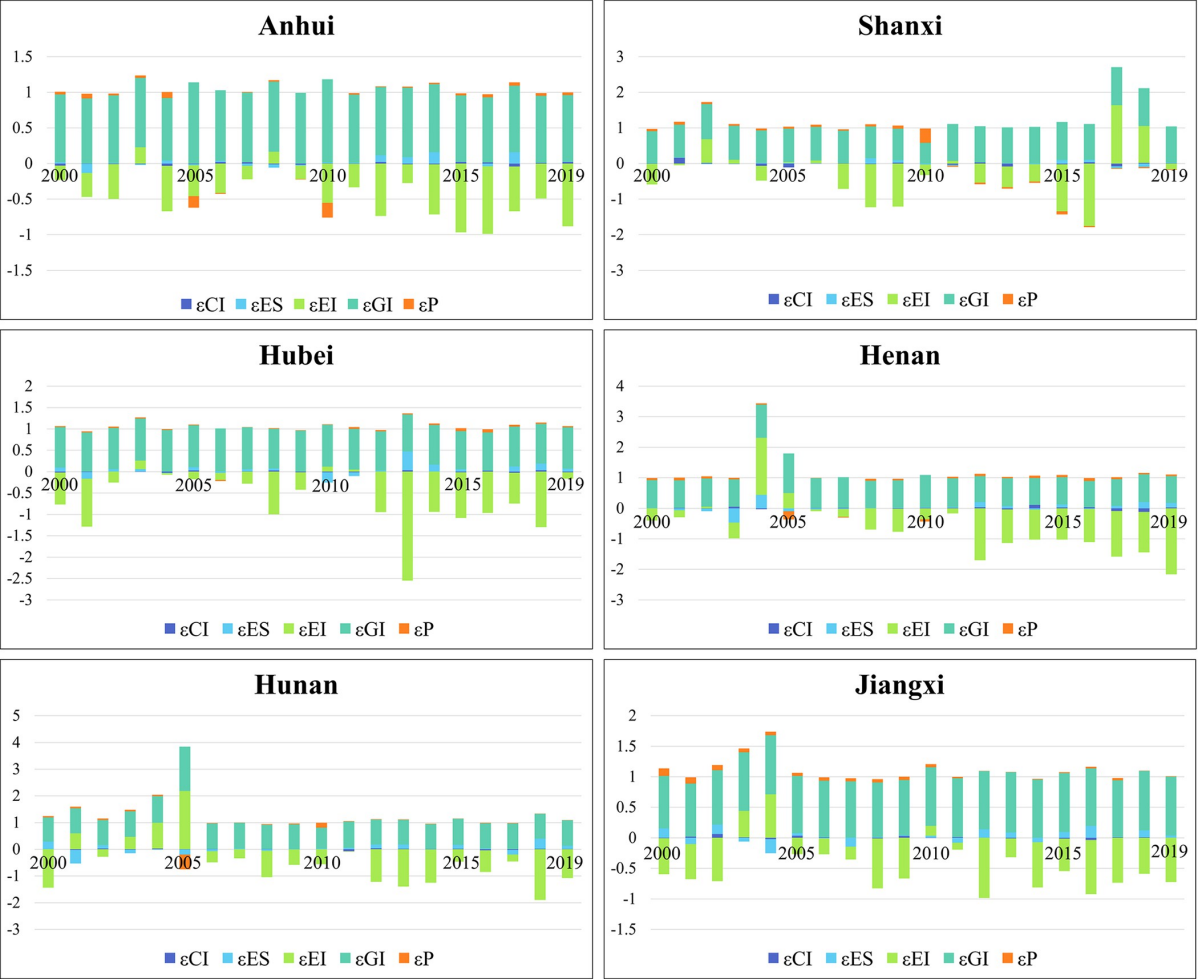
Decoupling status decomposition of six central provinces.

This paper decomposes the DI of the six central provinces into five factors: carbon emission decoupling factor (εCI), energy structure decoupling factor (εES), energy intensity decoupling factor (εEI), economic development decoupling factor (εGI), and population size decoupling factor (εP). Each driver’s absolute value reflects the degree of influence on the DI, with a positive value of the factor suggesting inhibition of the decoupling process and a negative value representing facilitation of the decoupling process. Fig 5 presents the decomposition results of the six central provinces from 2000–2019. In general, the factors GI and P are the key inhibitors of the decoupling process in the six central provinces. Conversely, the factor of EI acts as the primary driver, accelerating the decoupling process in each province. The influence of CI and ES factors fluctuates across the provinces.

From a provincial perspective, the decoupling performance of Anhui Province is influenced mainly by the inhibitory effect of GI and the promoting effect of EI. The GI consistently maintains an impact value of around 0.9, indicating a stable inhibitory effect. On the other hand, the EI shows an increasing trend, with its impact value increasing from 0.2 in 2000 to 0.88 in 2019. This demonstrates that Anhui Province has experienced improvements in technological efficiency and resource utilization. The decoupling process in Shanxi Province is predominantly influenced by GI and EI. GI exerts an inhibitory effect on decoupling, as evidenced by the numerical performance increasing from 0.906 in 2000 to 1.02 in 2019, with the inhibitory effect intensifying over time. Similarly, the impact of EI on the decoupling process in Shanxi Province is also notable. EI transitions from a facilitating effect to an inhibiting effect on decoupling performance post-2017. This implies a lag in technological innovation within the energy sector in Shanxi Province in recent years. Throughout this paper period, the inhibitory effect of GI on the decoupling process in Hubei Province remained consistently at approximately 0.9. Regarding promoting decoupling, EI showcased its highest impact in 2013, reaching a value of 2.55. However, this momentum was not sustained and stabilized over time, indicating that there is still ample room for further advancements through technological innovation in Hubei Province. The decoupling process in Henan Province is consistently influenced by GI, exhibiting a stable impact. Over the years, the promoting effect of EI on the decoupling has shown a progressive increase, ranging from -0.4 in 2000 to -2.16 in 2019. These findings suggest that Henan Province has made significant efforts toward technological innovation and prioritized optimizing its industrial structure in recent years [57]. The influence of GI on both Hunan Province and Jiangxi Province is similar, with values remaining around 0.9. However, the impact of EI on decoupling is more significant in Hunan Province than in Jiangxi Province. This suggests that the level of technological development in Hunan Province is more advanced than that in Jiangxi Province. As a result, Jiangxi Province could benefit from drawing on its neighboring province’s advanced experiences and practices.

In terms of sub-drivers, the EI and the GI have the most significant impact on decoupling the six central provinces. From 2000 to 2019, the EI performance showed fluctuations in the six central provinces, indicating that all provinces have made efforts toward technological progress. The promotion effect of EI on decoupling in the provinces of Anhui, Hubei, Henan, Hunan, and Jiangxi has been commendable. This suggests that these provinces have achieved advanced and comprehensive development in scientific and technological innovation, energy conservation, and environmental protection. Additionally, their industrial structure exhibits a reasonable combination of industries, resulting in higher energy utilization efficiency. However, the promotion effect of EI in Shanxi Province is relatively small and unstable. Due to the influence of historical development, the current EC and EG in Shanxi Province are still heavily reliant on coal. While technological progress has positively impacted improving EI, it has not reached the desired level of reducing overall EC. As a result, Shanxi Province should prioritize efforts toward industrial restructuring and the development of technological innovation. The impact of GI on the decoupling process performs similarly across provinces, with values remaining around 0.9. However, the performance of Shanxi Province is more remarkable, as the inhibitory effect of GI on the decoupling process is significant year after year, in contrast to the decoupling target. This effect has increased from 0.906 in 2000 to 1.02 in 2019, indicating that the current EG in Shanxi Province still heavily relies on EC. The CI, ES, and P decoupling factors have relatively less impact on the decoupling process observed in the province. Consequently, efforts should be made to transform the existing EG mode in the province.

### Prediction of decoupling status in six central provinces

The National Development and Reform Commission’s Energy Research Institute has produced three development models: high-speed, medium-speed, and low-speed, in accordance with its scenario-setting methodology. These models take into account changes in population size (PP), per capita GDP (PGI), energy intensity (PEI), and urbanization level (PU) et al. Considering provincial policies and central policies, the median value of population growth is used to represent the average level of future population size changes. A range of 0.6% -1.3% is set for population growth, with the high and median values reasonably set at ± 0.2%. The same approach is applied to set the values for per capita GDP growth rate, EI, and urbanization level growth rate, using historical data and considering the 14th five-year plan. The high and low values for each factor are reasonably set at ± 0.5%. Detailed information regarding the change rate settings of each factor can be found in Table 2.

**Table 2:** Rate of change setting for each factor.

Year	Rate of change	Population (%)	Per capita GDP (%)	Energy intensity (%)	Urbanization level (%)
2020–2025	High	1.30	5.00	-2.50	1.70
	Middle	1.10	4.50	-3.00	1.30
	Low	0.90	4.00	-3.50	1.00
2026–2030	High	1.20	4.00	-2.00	1.50
	Middle	1.00	3.50	-2.50	1.20
	Low	0.80	3.00	-3.00	0.95
2031–2035	High	1.10	3.00	-1.50	1.30
	Middle	0.90	2.50	-2.00	1.10
	Low	0.70	2.00	-2.50	0.90
2036–2040	High	1.00	2.00	-1.00	1.10
	Middle	0.80	1.50	-1.50	1.00
	Low	0.60	1.00	-2.00	0.85

The multivariate regression of the six central provinces model detected the existence of multicollinearity among the variables. This paper used ridge regression on the six models to eliminate the influence of multicollinearity. The results of ridge regression were obtained, as shown in Table 3. Subsequent testing confirmed a good fit between the actual values of CE and the predicted values from 2000 to 2019. This suggests that it is indeed feasible to use these six models to predict future CE in the six central provinces.

**Table 3:** Ridge regression treatment results.

Province	K value	Equation	R^2^	F
Anhui	0.195	LnCE1 = 60.821+0.208 × LnPGI1–4.438 × LnPP1–0.250 × LnPEI1 + 0.627 × LnPU1	0.966	108.138 (0.000***)
Shanxi	0.113	LnCE2 = 0.031+0.384 × LnPGI2+2.434 × LnPP2+0.361 × LnPEI2 + 0.779 × LnPU2	0.959	88.632 (0.000***)
Hubei	0.049	LnCE3 = 52.916+0.508 × LnPGI3–4.069 × LnPP3+0.353 × LnPEI3 + 0.961 × LnPU3	0.951	72.585 (0.000***)
Henan	0.098	LnCE4 = 72.941+0.453 ×LnPGI4–5.857 × LnPP4 + 0.345 × LnPEI4+0.869 × LnPU4	0.956	81.965 (0.000***)
Hunan	0.062	LnCE5 = 39.926+0.480 ×LnPGI5–2.592 × LnPP5+0.515 × LnPEI5+1.175 × LnPU5	0.973	137.351 (0.000***)
Jiangxi	0.094	LnCE6 = - 35.255+0.222 × LnPGI6+6.780 × LnPP6+0.073 × LnPEI6+0.547 × LnPU6	0.985	252.090 (0.000***)

Note

*** Represents significant at the 1% level.

**Fig 6:**
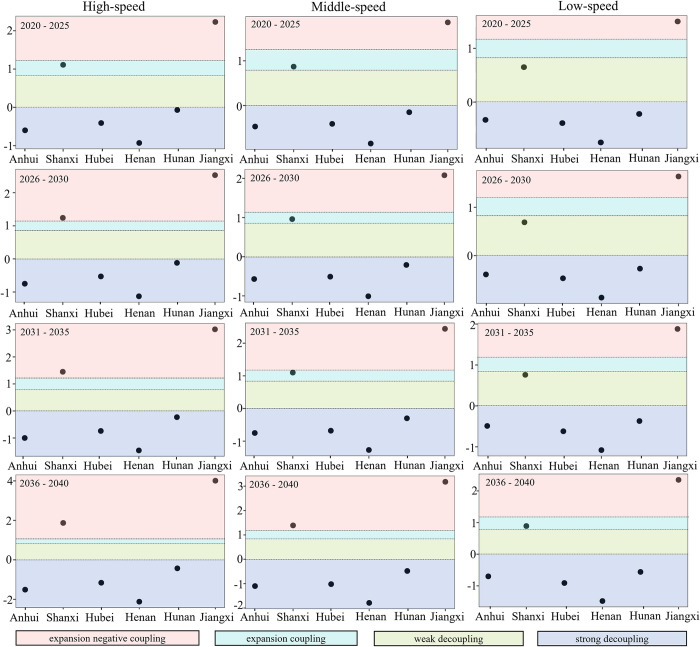
Decoupling status forecast of six central provinces.

According to the prediction equations in this paper, combined with the set values of the growth rates of each variable, the decoupling status in the period 2020–2040 is predicted, as shown in Fig 6. During 2020–2040, Anhui, Hubei, Henan, and Hunan provinces will demonstrate stable and robust decoupling under the high-speed, medium-speed, and low-speed development scenarios. These provinces are able to achieve the decoupling targets while also meeting the Dual Carbon Goals.

Beginning in 2020, the Anhui, Hubei, Henan, and Hunan provinces can all achieve strong decoupling. Anhui Province is predicted to double its GDP per capita by 2040, from 37,000 to 75,000 CNY per person, due to a multitude of policies put in place to benefit the people of Anhui Province. It is also worth noting that Anhui’s population will remain relatively high over the next 20 years, indicating the efficiency of the province’s measures in attaining economic development while meeting the decoupling aim. Middle and low-development rates react similarly to high-development rates. Over the next 20 years, Hubei Province will progressively raise its urbanization rate while decreasing its EI. It suggests that the province’s efforts to develop new technologies will significantly contribute to attaining the Dual Carbon Goals. Henan Province’s EI is predicted to fall to about 0.5 by 2040, indicating that the province has a significant incentive to innovate in energy technology. Hunan Province’s EI decrease is reduced from 0.6 to around 0.4, and it must focus on technical innovation in energy conservation and carbon reduction.

**Fig 7:**
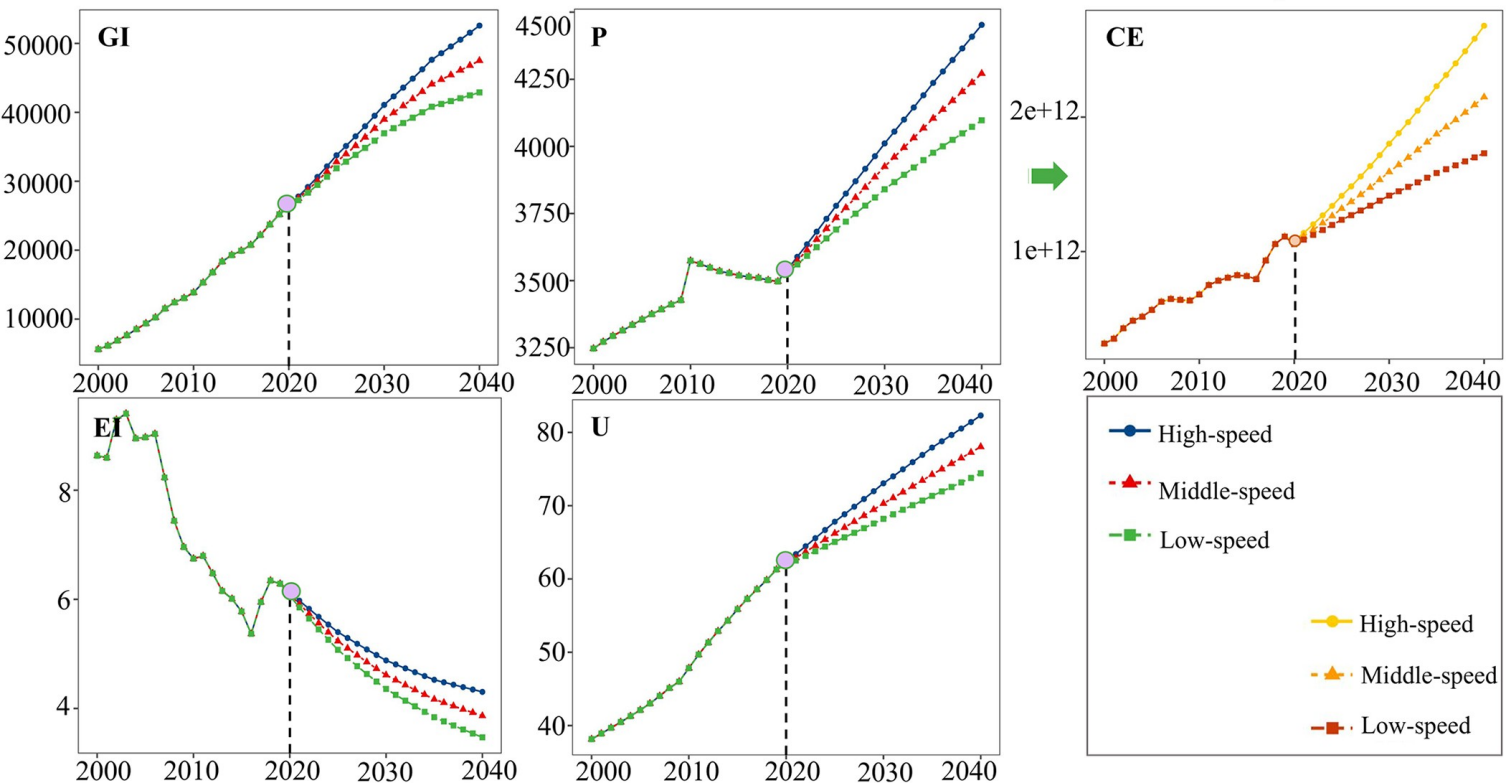
Projected indicators for Jiangxi Province.

However, the decoupling performance of Shanxi and Jiangxi provinces is unsatisfactory, particularly in Jiangxi province. Assuming the policy’s development pace is followed, Jiangxi Province will experience Reverse Decoupling between 2020 and 2040. As shown in Fig 7, despite reducing EI, Jiangxi Province’s CE continues to demonstrate a near-expanding increase under the high-speed scenario. This suggests that Jiangxi Province can develop at a different rate than other provinces. It needs to establish development targets based on its circumstances, considering limitations imposed by historical development conditions and other factors. Due to these constraints, Jiangxi Province must set development goals tailored to its specific situation and strive to achieve CE and EG decoupling as soon as possible.

**Fig 8:**
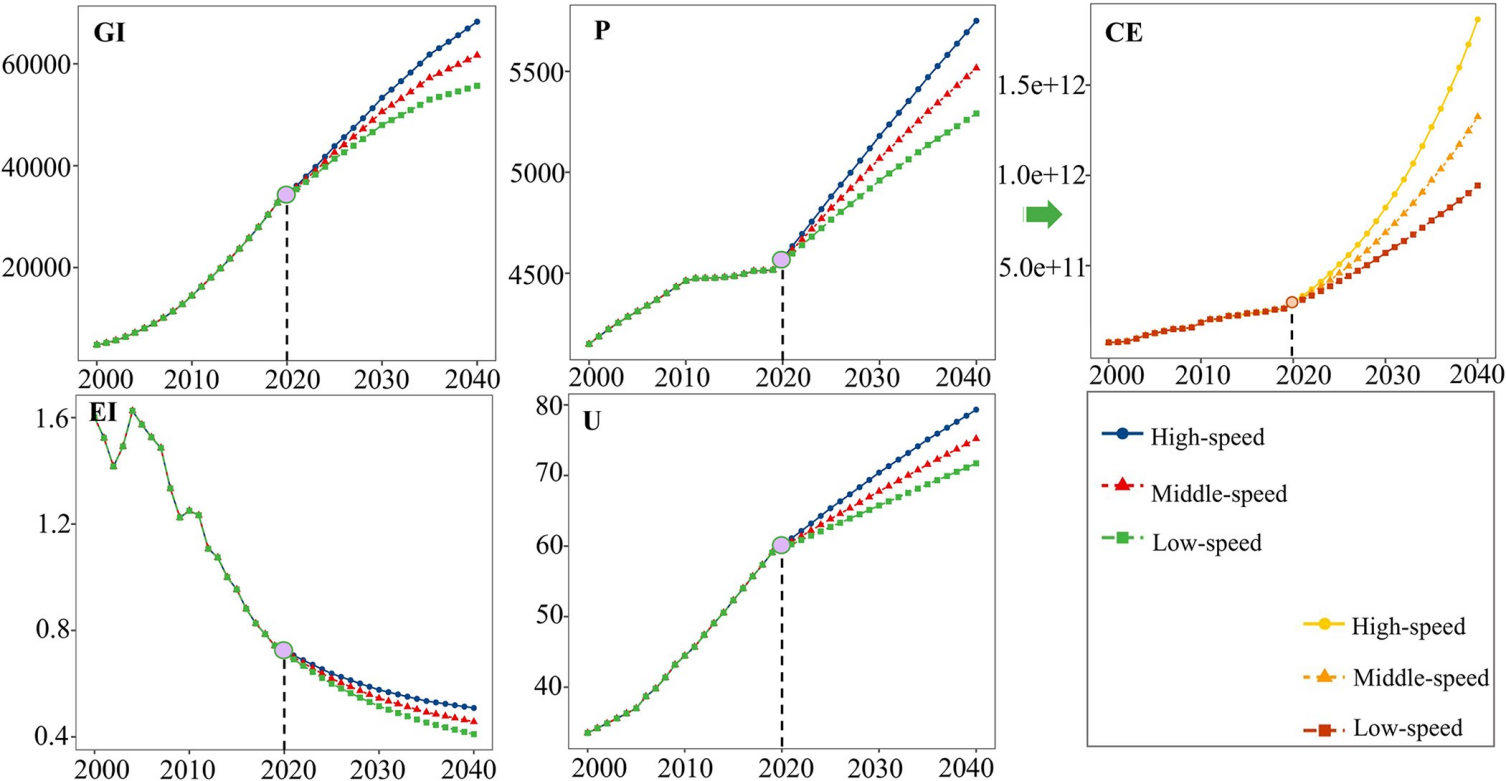
Projected indicators for Shanxi Province.

As shown in Fig 8, CE in Shanxi Province will increase significantly in the next 20 years. Shanxi province exhibits negative expansion coupling and expansion coupling under high-speed and medium-speed development. Only weak decoupling under low-speed development. Different rates of development play an important role in achieving decoupling. Therefore, the rapid rate of EG in Shanxi province is not conducive to the decoupling of CE and EG. This is mostly because Shanxi is a major coal province, and its economy is highly reliant on coal consumption. As a result, the demand for coal will rise significantly if rapid economic expansion is required in a short time. To fulfill the Dual Carbon Goals without increasing the need for coal, Shanxi Province must carefully assess the rate of EG. It is important to keep the target of EG within a reasonable range.

In summary, the decoupling performance of the six central provinces from 2020 to 2040 can be considered satisfactory, generating hopes for the achievement of the Dual Carbon Goals. However, Shanxi and Jiangxi Provinces need to pay special attention to setting their economic goals, considering their own development conditions and limitations. They need to set reasonable development goals and not blindly pursue rapid development. Otherwise, it will substantially impede overall progress toward the Dual Carbon Goals.

## Discussion and policy recommendations

### Discussion

Based on the EC dataset of six central provinces in China from 2000 to 2019, this paper first analyzes the decoupling status between CE and EG using the Tapio decoupling model. Subsequently, the DI is decomposed into five decoupling drivers, namely CI, ES, EI, GI, and P, utilizing the LMDI method. At last, an improved STIRPAT model is employed to analyze the decoupling status of CE and EG in the six central provinces from 2020 to 2040 through overall scenario analysis. It provides a certain reference value for designing policies to achieve Dual Carbon Goals.

From China’s 10th five-year period to the 13th five-year period, the decoupling status of CE from EG in the six central provinces exhibited an upward trajectory. Stable decoupling did not occur before 2005. However, with the implementation of the Rise of Central China Strategy in 2006, the overall decoupling performance of the six central provinces began to improve consistently. This improvement can be attributed to technological innovation and optimization of the industrial structure, which has facilitated the decoupling process in these provinces. This phase not only encouraged the EG of the six central provinces but also hastened the process of achieving the aim of decoupling. This is consistent with the findings of Gao et al. and Chen et al., which once again demonstrate the quality and validity of the paper [2,58]. This research also considers the impact of ES, EI, P, and other factors on CE from the perspective of EC. This gives the six central provinces with high CE a more comprehensive perspective on the decoupling of EG and CE, allowing them to draw more accurate conclusions and provide better direction to the government when developing policies.

The impact of decoupling variables on each province varies significantly, resulting in a lack of harmony in the growth of the northern and southern provinces. From a macro perspective, the decoupling process in each province is primarily driven by EI while being hindered by GI. From a micro-perspective, the five decoupling factors influence the provinces to varying degrees and in different directions. The economic agglomeration effect in the six central provinces is similar. The decoupling effect of the GI factor remains around 0.9 for each province. The increase in EI strongly contributes to the decoupling process in the economically significant provinces of Henan and Hunan but has a weak contribution to the decoupling of the other four provinces. The impact of the GI is evident in Shanxi and Henan provinces, with the coefficients varying from -0.1 to 0.159 in Shanxi province and -0.114 to 0.113 in Henan province, which is consistent with the findings of Chun et al. [57]. This indicates that the unstable development of the EI in these two provinces resulted in significant changes in the coefficients of CE and EC. The ES factor fluctuates between positive and negative values for each province, implying that it does not significantly contribute to the decoupling process.

The STIRPAT model was developed using scenario analysis to study the changes in the decoupling status of the six central provinces from 2020 to 2040. The results show significant differences among the six provinces. Anhui, Hubei, Henan, and Hunan show significantly strong decoupling, with decoupling indices distributed between -2.21 and -0.07 in all three scenarios. This demonstrates that the four provinces can all achieve decoupling as expected. However, Shanxi and Jiangxi provinces have behaved extremely unusually. The Reverse Decoupling status occurs when Shanxi Province develops at middle or high speed, suggesting that low-speed development is more suitable for Shanxi Province. The performance of Jiangxi Province is even more unusual, as none of the three development rates can be decoupled during the 2020–2040 period. This differs from previous research, since Fang et al.’s analysis shows that carbon emissions in Shanxi Province would peak between 2026 and 2030, while Jiangxi Province will likewise experience a carbon peak between 2036 and 2040 [10]. However, the findings of this analysis suggest that it will be difficult for Shanxi and Jiangxi provinces to achieve decoupling status between 2020 and 2040. This clearly demonstrates that there is no apparent connection between attaining carbon peaking and realizing decoupling. As we all know, the Dual Carbon Goals is not to prohibit carbon emissions but to limit them within a specified range, and the primary goal of such a target is to encourage economic growth while reducing carbon emissions. Therefore, the measurement of provinces to achieve decoupling status is more meaningful for the study of Dual Carbon Goals. This paper shows that the development of Jiangxi has opened a significant distance from the other five provinces. If Jiangxi Province does not accelerate in bridging the gap, it will not be conducive to completing the Rise of Central China Strategy.

### Policy recommendations

To fully promote the decoupling of EG and CE in the six central provinces under the Dual Carbon Goals, it is necessary to re-examine each province’s development advantages and establish development paths tailored to the province’s characteristics. Based on the research findings and simulation results, this paper proposes the following approaches to help achieve the decoupling target.

Firstly, the decoupling performance of the six central provinces differs significantly north-south, with a mismatch between economic agglomeration and other development drivers. Hence, the optimization of the ES is necessary in each province. Besides, the P has a discernible negative impact on decoupling, especially in the densely populated region of Henan [57]. In the next stage, Henan Province should focus on attracting talent and promoting population agglomeration. The main development objective of Shanxi and Jiangxi Provinces in the next stage is to strongly advocate technological innovation and optimize the ES through technological development.

Secondly, the development targets for the six central provinces do not apply to Shanxi and Jiangxi provinces. It is necessary to set development targets considering regional differences. According to the 2020–2040 decoupling projections, all four provinces, except for Shanxi and Jiangxi, demonstrate stable and positive decoupling under the three basic scenarios. The six central provinces’ economic cooperation should be strengthened in the framework of the new international and domestic double-cycle development environment. Therefore, Hubei Province should take the lead in capitalizing on its geographical location and serving as a model for inter-provincial cooperation. Wuhan is the center metropolis of the six central provinces, with a strong development endowment and momentum, and its radiation-driven function in adjacent provinces should be strengthened [59]. Jiangxi Province should prioritize scientific and technological innovation in the development process and promote high-quality development through technological innovation. Shanxi Province, as the energy revolution’s forerunner, should hasten the release of high-quality coal production capability and its practical application. Take several steps to promote Shanxi Province’s low-carbon transformation and decoupling goals. Furthermore, the importance of cities should be stressed [60]. Pay attention to the radiation-driven roles of Zhengzhou, Changsha, Hefei, and so on.

Thirdly, in the context of regional coordinated development integration, the six central provinces should rationally establish their development targets, taking into account their resource endowments and historical circumstances. This will enable the scientific and efficient realization of the decoupling plan in accordance with their respective development situations. To promote inter-provincial collaboration, each province should focus more on the growth of its specialized industries. Advantages differ by province. As significant science and education provinces, Hubei and Hunan have more potential for scientific and technological innovation development. As a populous province, Henan province should take full advantage of its demographic advantages. Talent upgrading will result in the rejuvenation and iteration of labor-intensive sectors. Anhui Province continues to enhance its scientific and technological capabilities in areas such as energy storage and carbon capture. Shanxi and Jiangxi provinces should focus on the Dual Carbon Goals, deepen institutional reform in the energy industry, and choose a development path that fits the province’s unique characteristics.

## Conclusion

EC is a fundamental pillar of EG and plays a critical role in societal development. However, EC has resulted in a massive amount of CE, creating significant contamination of the environment. CE from EC in the six central provinces accounts for a sizable part of the total national CE. Therefore, it is particularly crucial to analyze the relationship between CE and EG in the six central provinces.

China’s CE of EC increased from 1054 Mtc in 2000 to 3168 Mtc in 2019, with an average yearly growth rate of 5.7%. Among the six central provinces, Shanxi province had the highest CE, closely followed by Henan, Anhui, Hubei, Hunan, and Jiangxi provinces. Meanwhile, China’s GDP maintained a growth rate of around 10% between 2000–2019. This research uses the Tapio model, a classical decoupling analysis model, to investigate the decoupling status between CE and EG in the six central provinces from 2000 to 2019. The results demonstrate that the six central provinces as a whole are in a condition of decoupling, although the performance of decoupling differs greatly between provinces. This presents a significant challenge for the six central provinces in implementing the Rise of Central China strategy in a coordinated and integrated way. The research then decomposes the primary elements influencing the status of decoupling using the LMDI model, which is better suited to solving the zero-value problem. The findings revealed that decoupling proceeded primarily through the inhibitory effects of GI and P, as well as the facilitating influence of EI. Factors have a different impact in each province. The research finishes by utilizing the STIRPAT model to anticipate the decoupling status of the six central provinces over the next two decades from a novel perspective. The results reveal that, with the exception of Shanxi and Jiangxi provinces, the other four provinces can achieve a significant decoupling state, which challenges the findings of earlier research that has undertaken carbon peaking forecasts. As a result, in the context of coordinated development, the six central provinces should focus on combining their strengths and compensating for their developmental deficiencies, optimizing their ES and increasing their EI, and setting development goals that are appropriate for them.

Although this study achieved certain theoretical and practical results, there are some weaknesses that must be addressed further due to the complexity and systematic nature of the research subject. Future research could be expanded in the following areas.

On the one hand, due to the timing of data updates, this study solely examined the decoupling status of the six central provinces between 2000 and 2019. In future investigations, fresh and more extensive data can be employed for testing to improve result accuracy. On the other hand, this study only examined the decoupling status and affecting factors of each province separately, and spillovers between provinces should be further considered. It is necessary to investigate their mutual influences, mechanisms, and mediating effects.
